# Differential Lipid Signatures of Lumbar and Cisternal Cerebrospinal Fluid

**DOI:** 10.3390/biom14111431

**Published:** 2024-11-11

**Authors:** Trine L. Toft-Bertelsen, Søren Norge Andreassen, Nicolas H. Norager, Anja Hviid Simonsen, Steen Gregers Hasselbalch, Marianne Juhler, Nanna MacAulay

**Affiliations:** 1 Department of Neuroscience, University of Copenhagen, 2200 Copenhagen, Denmark; 2 Department of Neurosurgery, Neuroscience Centre, Copenhagen University Hospital-Rigshospitalet, 2200 Copenhagen, Denmark; 3 Danish Dementia Research Centre, Department of Neurology, Section 6911, Copenhagen University Hospital-Rigshospitalet, 2200 Copenhagen, Denmark; 4 Department of Clinical Medicine, University of Copenhagen, 2200 Copenhagen, Denmark

**Keywords:** CSF, lipidomics, mass spectrometry, biomarkers

## Abstract

Background: The molecular composition of cerebrospinal fluid (CSF) is often used as a key indicator of biochemical alterations within distinct brain and spinal cord fluid compartments. The CSF protein content in lumbar CSF samples is widely employed as a biomarker matrix for diagnosing brain-related pathological conditions. CSF lipid profiles may serve as promising complementary diagnostics, but it remains unresolved if the lipid distribution is consistent along the neuroaxis. Methods: The lipid composition was determined with liquid chromatography coupled with tandem mass spectrometry (LC-MS/MS) in cisternal CSF obtained from healthy subjects undergoing preventive surgery of an unruptured aneurism (*n* = 11) and lumbar CSF obtained from individuals referred for the clinical evaluation of cognitive dysfunction but subsequently cleared and deemed healthy (*n* = 19). Results: We reveal discernible variations in lipid composition along the neuroaxis, with a higher overall lipid concentration in cisternal CSF, although with different relative distributions of the various lipid classes in the two compartments. The cisternal CSF contained elevated levels of most lipid classes, e.g., sphingomyelins, lysophosphatidylcholines, plasmenylphosphatidylcholines, phosphatidic acids, and triacylglycerols, whereas a few select lipids from the classes of fatty acids, phosphatidylcholines, amides and plasmenylphosphatidylethanolamines were, oppositely, elevated in the lumbar CSF pool. Conclusions: The distinct lipid distribution along the neuroaxis illustrates that the molecular constituents in these two CSF compartments are not uniform. These findings emphasize the necessity of establishing a lumbar lipid index for the accurate interpretation of the cranial CSF lipid profile.

## 1. Introduction

Cerebrospinal fluid (CSF) surrounds the brain and fills the ventricles in the brain. CSF is in continuum with the interstitial fluid [1,2], and its molecular composition thus serves as an indicator for biochemical alterations occurring within various CSF compartments. CSF has garnered considerable attention as an invaluable biomarker matrix for diagnosing pathological conditions afflicting the brain with special attention to proteomics, metabolomics, and multi-omics delineation of inflammatory markers [3,4,5,6,7]. Lipidomics have been somewhat marginalized in these research pursuits, despite the brain’s distinction as the organ bearing the highest concentration of lipids [8], encompassing diverse lipid classes such as fatty acids, phospholipids, sphingolipids, glycerolipids, and sterols [8]. Lipids, constituting a fundamental class of cellular constituents, play a multifaceted role in various critical cellular processes. Their involvement spans cell differentiation, proliferation, survival, and programmed cell death [9], and as such, strict regulation of lipids is anticipated. Notably, disturbances in the equilibrium of various lipid species have been observed in patients suffering from subarachnoid hemorrhage [4,10,11] and traumatic brain injury [12], as well as in an animal model of intraventricular hemorrhage (IVH) [11]. Consequently, lipids identified in CSF hold potential as valuable biomarkers for disease, as a screening tool, as an additional resource for diagnostic investigations as seen for other molecules [4,13,14,15,16,17,18], or potentially for disentangling the underlying etiology of various brain pathologies, i.e., posthemorrhagic hydrocephalus (PHH) [11]. The field of lipidomics, which entails a comprehensive analysis of lipids within specific compartments, is uniquely suited for evaluating CSF lipid imbalances associated with brain-related disorders. However, there is limited research on the comprehensive lipid composition of CSF in healthy individuals with only one study reporting a full lipidomic signature in CSF collected from the ventricles [4]. Accessing human cisternal or ventricular CSF is primarily feasible during neurosurgical interventions for intracranial diagnostic purposes or the management of neurological disorders. In contrast, lumbar puncture, the standard method for obtaining CSF samples for diagnostic purposes, is widely employed across various clinical scenarios with several prevalent neuropathologies (e.g., Alzheimer’s disease, multiple sclerosis) [6,19,20,21,22,23] when diagnostic CSF analysis is an integral component of clinical evaluation. This practice stems from the technical ease and ethical considerations associated with lumbar CSF collection. Access to the intracranial compartments is assumed to offer a more accurate reflection of brain pathology, but it is much more invasive with a severe burden of potential complications. Thus, it is crucial to recognize that the lumbar CSF compartment is anatomically distinct from the ventricular compartment [5,24,25,26,27,28], which gives rise to a potential gradient in lipid concentration along the neuroaxis. This compartmentalized distribution of various biomarkers thus presents a significant challenge to the field of CSF diagnostics in which it remains unclear whether lipid biomarkers identified in lumbar CSF can reliably reflect intracranial pathology.

In this study, we aimed to elucidate differences in lipid composition between the two CSF compartments by resolving the lipid composition in both cisternal and lumbar CSF, by liquid chromatography coupled with tandem mass spectrometry (LC-MS/MS). This technique represents a highly precise analytical platform for untargeted lipidomics. Our findings revealed discernible variations in CSF lipid compositions between these two compartments, underscoring the importance of refraining from directly extrapolating lipid levels from lumbar CSF samples to intracranial CSF. Accordingly, we propose the establishment of a lumbar lipid index.

## 2. Methods

### 2.1. Patients

Cisternal CSF samples were collected between June 2019 and September 2021 from 11 control subjects (average 64 years, range 40–77 years, 7F/4M) undergoing preventive surgery for unruptured aneurysms (vascular clipping). The CSF was collected from the basal cisterns during surgery before clipping of the aneurysm. CSF samples obtained from subjects within the same cohort have previously been analyzed for lipid signatures in comparison to CSF obtained from patients with subarachnoid hemorrhage [4]. Lumbar CSF samples were collected from 19 individuals (average 75 years, range 65–84 years, 7F/12M) who were referred for evaluation based on suspicion of cognitive dysfunction but did not fulfill the criteria for dementia, cognitive impairment, or neurodegenerative diseases based on clinical, cognitive, and neuroimaging clinical evaluation. CSF samples were obtained as part of the diagnostic procedure. CSF samples obtained from subjects within the same cohort have previously been analyzed for lipid signatures in comparison to CSF obtained from patients with normal pressure hydrocephalus [29]. All CSF samples were centrifuged at 2000× *g* for 10 min at 4 °C within 2 h from collection and subsequently stored at −80 °C. Written informed consent was obtained from all patients or next of kin depending on the capacity of the patients. The study abides by the Declaration of Helsinki principles and was approved by the Ethics committee of the Capital Region of Denmark (H-19001474/69197, H-17011472, and H-18046630) and Danish Data Protection Agency (VD-2019-210).

### 2.2. Liquid Chromatography with Mass Spectrometry (LC-MS) Analysis

To precipitate proteins, samples were mixed with isopropanol containing 0.1 M butylated hydroxytoluene and stable isotope-labeled internal standards and subsequently filtrated using Spin-X filters (Avantor, vwr) (1400 rpm at 5 °C for 2 min). Following, the sample was transferred into a high-recovery HPLC vial with eluent mix and capped. Liquid chromatography-mass spectroscopy (using ultra-high pressure liquid chromatography instruments coupled with Thermo Scientific Vanquish LC (Waltham, MA, USA) coupled to Thermo Q Exactive HF mass spectrometers) was used as an untargeted analysis of the CSF samples and conducted by MS-Omics, Denmark. Ionization was performed in positive and negative ionization mode using an electrospray ionization interface. The chromatographic lipids were separated on a Waters^®^ ACQUITY Charged Surface Hybrid (CSH™) C18 column (2.1 × 100 mm, 1.7 µm). The column was thermostated at 55 °C. The mobile phases consisted of (A) acetonitrile/water (60:40) and (B) isopropanol/acetonitrile (90:10), both with 10 mM ammonium formate and 0.1% formic acid. Lipids were eluted in a two-step gradient by increasing B in A from 40 to 99% over 18 min. The flow rate was 0.4 mL/min. The MS was operated with the following settings: *m/z* range: 200–1500; AGC target: 1 × 10^6^; Resolution: 120,000; collision energy: 30. Compounds were extracted based on features (a peak characterized by one mass and one retention time) and additional information (e.g., isotope pattern, accurate mass, and molecular formula). The retention time of compounds within the same lipid class is estimated using the relation between retention time and the chain length and number of double bonds. Three levels of annotation were used (level one–three) with level one representing the most confident identifications (based on accurate mass, tandem mass spectrometry spectra (MSMS) and estimated retention time), level two, based on two of these sets of information, is divided into two sublevels; accurate mass and estimated retention, and level three is based on library searches using the accurate mass and elemental composition alone. In this study, level three annotations were not included. As a quality control, a mixed pool sample was created by taking a small aliquot from each sample to be analyzed with regular intervals through the sequence. The quality control is based on spikes in stable isotope-labeled internal standards, pooled QC samples, and blank injections. The internal standards are used to monitor instrument performance (retention time stability and mass accuracy) over all injected samples, while the pooled QC samples are used to evaluate the performance of the detected compounds. The final dataset consists of compounds that have passed a range of quality criteria: S/N > 5, precision < 20% (calculated as relative standard deviation across pooled QC samples), correlation between signal and dilution factor of QC > 0.8, and ratio of the average signal in pooled QC samples and average signal in client samples >0.5 and <2 (Supplementary Figure S1). Splash lipidomix (Avanti 330707), a single-vial prepared lipidomic analytical standard for human plasma lipids, was diluted 1:50 as prescribed by the manufacturer and used as internal standards for QC check (retention time, intensity, and mass accuracy) to ensure that all samples were analyzed correctly. The mixture components were 15:0-18:1(d7)PC (150.6 µg/mL), 15:0-18:1(d7)PE (5.3 µg/mL), 15:0-18:1(d7)PS (3.9 µg/mL), 15:0-18:1(d7)PG (26.7 µg/mL), 15:0-18:1(d7)PI (8.5 µg/mL), 15:0-18:1(d7)PA (6.9 µg/mL), 18:1(d7)LPC (23.8 µg/mL), 18:1(d7)LPE (4.9 µg/mL), 18:1(d7)cholesterol (329.1 µg/mL), 18:1(d7)MG (1.8 µg/mL), 15:0-18:1(d7)DG (8.8 µg/mL), 15:0-18:1(d7)-15:0TG (52.8 µg/mL), 18:1(d9)SM (29.6 µg/mL), and cholesterol(d7) (98.4 µg/mL). Data were processed using Compound Discoverer 3.0 (ThermoFisher Scientific, Waltham, MA, USA).

### 2.3. Bioinformatics and Statistical Analyses

A Smirnov–Grubbs test (two-sided, α = 0.05) was performed for all detected compounds (358) within each group. The number of outliers was counted for each sample, and samples were excluded if a sample presented with more than 20% outliers. All 358 compounds were manually curated into 35 main classes of lipids, non-biological lipid compounds, and non-classified (‘others’). The latter two groups were excluded from the analysis. The ‘small group collection’ contained less than four lipids (<1% of the total detected compounds). The remaining 17 groups with 244 compounds were employed for the final analysis. Principal component analysis (PCA) plots [30] were employed to detect separation of the two cohorts and were generated with the normalized lipid values for each sample (based on the geometric mean) or displayed within each lipid group. All quantifications in the volcano plot and the bar plots were generated using normalized data without outliers (two-sided Smirnov–Grubbs test, α = 0.05). Statistical tests were conducted with the Welch’s *t*-test followed by the Benjamini–Hochberg method (with an adjusted *p* value < 0.05 (false discovery rate, FDR, of 5%)) [31,32]. All analysis scripts can be found at https://github.com/Sorennorge/MacAulayLab-Cisternal-Lumbar-Metabolomics.

## 3. Results

### 3.1. Differential CSF Lipid Signature in Cisternal and Lumbar CSF

**Figure 1:**
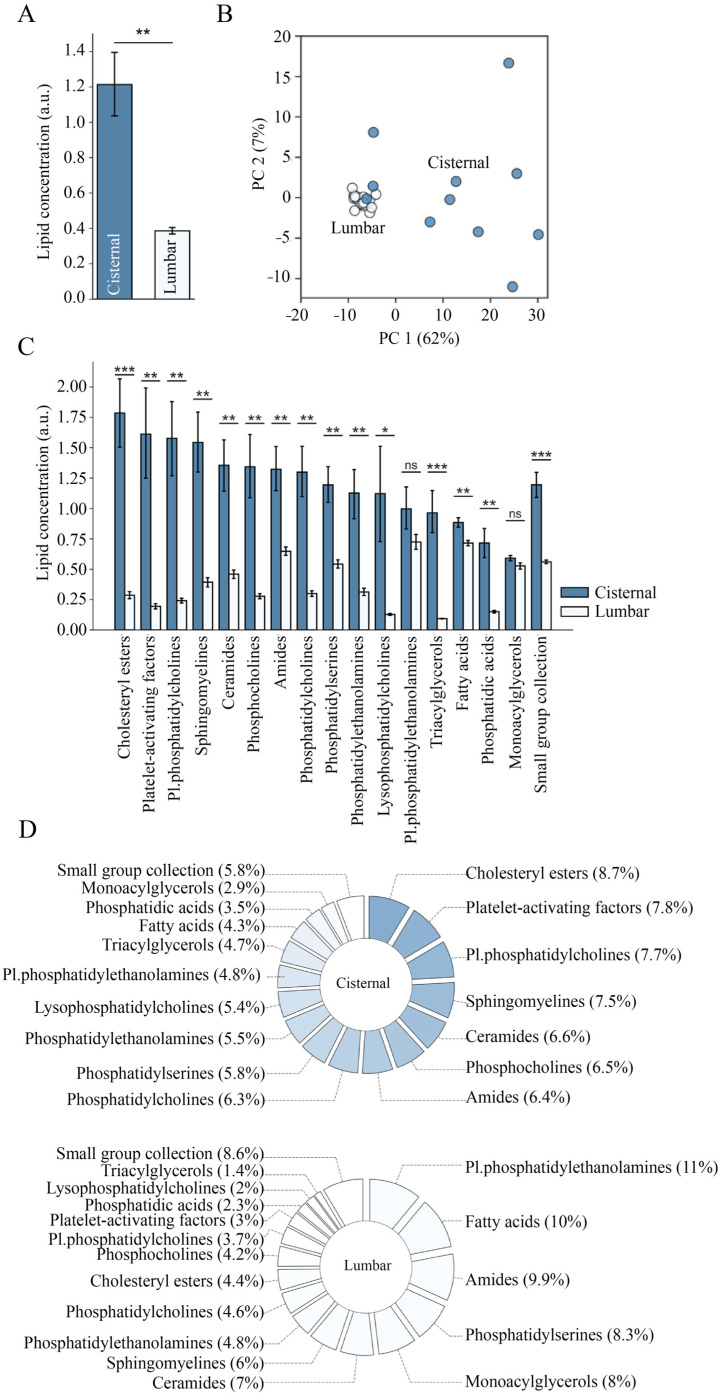
Distinct lipid signatures in cisternal CSF vs. lumbar CSF. (**A**) The total lipid concentration in cisternal and lumbar CSF. (**B**) The total lipid values were grouped for cisternal CSF and lumbar CSF and plotted in a PCA plot. (**C**) The amount of each lipid class in cisternal and lumbar CSF. (**D**) The relative distribution of the various lipid classes in the two CSF compartments. Data are based on lumbar CSF from 19 control subjects and cisternal CSF from 11 control subjects. Statistical evaluation was conducted with Welch’s t-test. *; *p* < 0.05, **; *p* < 0.01, ***; *p* < 0.001, ns; not significant.

CSF was obtained from healthy individuals: (1) lumbar CSF from patients undergoing diagnostic evaluation for neurodegenerative diseases or NPH but later found not fulfilling the diagnostic criteria for either; (2) CSF from the basal cisterns sampled during preventive clipping of an unruptured aneurism in otherwise healthy individuals. Mass spectroscopy-based lipid quantification detected 358 compounds of which 244 were classified as lipid compounds distributed across 17 lipid classes with those consisting of fewer than four lipids (<1%) combined and termed ‘small group collection’ (Supplementary Table S1, Sheet 1). The lipid concentration was ~3 fold higher in cisternal CSF compared to that obtained from the lumbar compartment (0.39 ± 0.01 a.u., *n* = 19 in lumbar CSF vs. 1.21 ± 0.19 a.u., *n* = 11 in cisternal CSF, *p* < 0.01), as shown in Figure 1A. In addition, a principal component analysis (PCA) of the CSF lipid content revealed a distinct overall lipid composition in these two CSF compartments (Figure 1B). PCA performed on the individual lipid classes demonstrated that these were mostly distributed in a similar manner although with a varying extent of compartmentalization (Supplementary Figure S2).

### 3.2. Lipids Groups Are Upregulated in Cisternal CSF Compared to Lumbar CSF

To identify individual lipid classes that may drive the diversion of the overall lipid profiles in cisternal vs. lumbar CSF from healthy subjects, we compared the concentration of different lipid classes between the two CSF compartments. All lipid classes, except monoacylglycerols and plasmenylphosphatidylethanolamines, were significantly more abundant in cisternal CSF (Figure 1C). This cisternal elevation of lipids may originate in the overall increased concentration of lipids in this compartment. The relative distribution of the various lipid classes in the two CSF compartments is illustrated in pie charts (Figure 1D) that reveal cholesteryl esters (8.7%), platelet-activating factors (7.8%), plasmenylphosphatidylcholines (7.7%), sphingomyelines (7.5%) and ceramides (6.6%) as the five most represented lipid classes of the 17 classes in total in cisternal CSF, whereas plasmenylphosphatidylethanolamines (11%), fatty acids (10%), amides (9.9%), phosphatidylserines (8.3%) and monoacylglycerols (8.0%) were the five most represented lipid classes in lumbar CSF. The remaining classes likewise distributed differently within the CSF pools.

### 3.3. Select Lipids Are Upregulated in Each of the CSF Compartments

**Figure 2:**
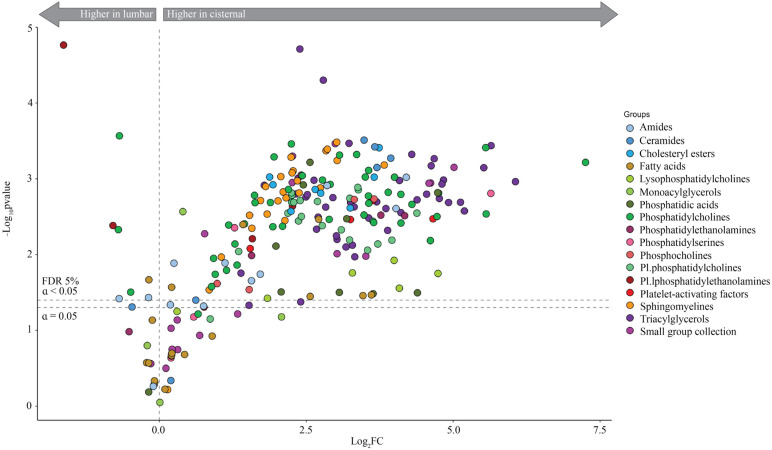
Lipid class analysis. Volcano plot of individual CSF lipids identified with the fold change (log_2_ transformed) between cisternal CSF and lumbar CSF. The upper dashed line indicates cut-off for significance (at a false discovery rate, FDR, <0.05). FC: fold change. Data are based on lumbar CSF from 19 control subjects and cisternal CSF from 11 control subjects. Statistical evaluation was conducted with Welch’s t-test followed by the Benjamini–Hochberg method.

**Figure 3:**
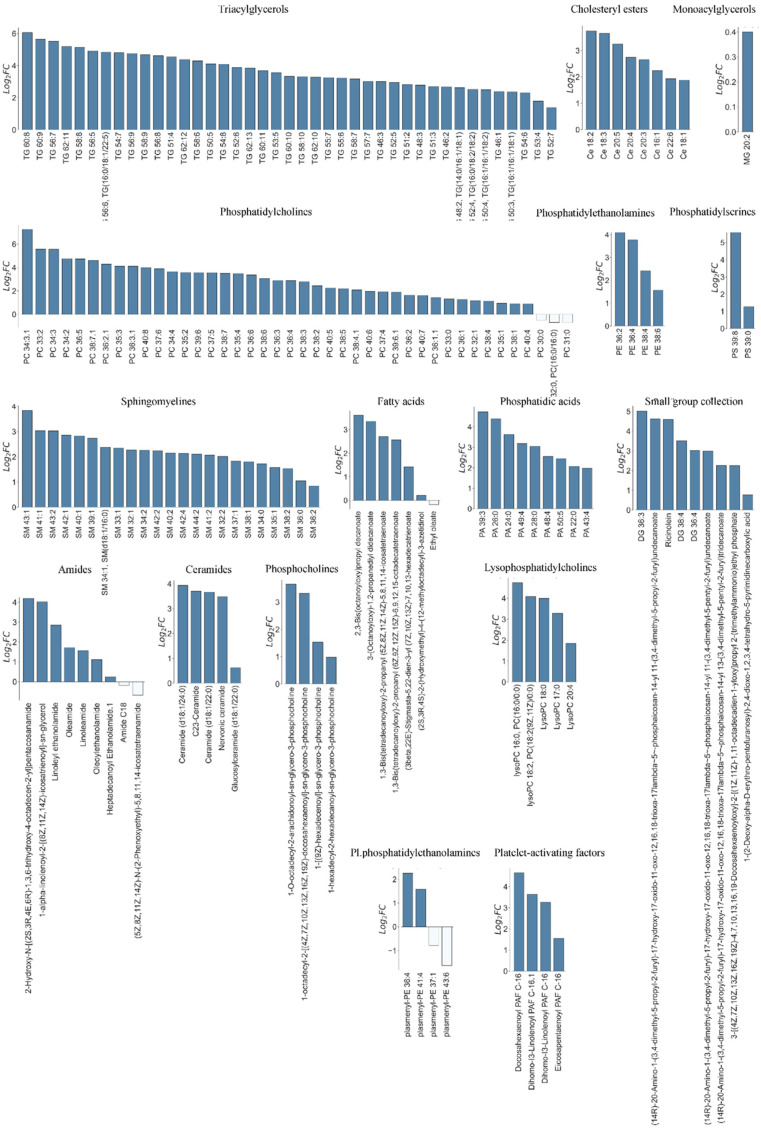
Isolated lipid class analysis. The lipid content within each lipid class in isolation between cisternal and lumbar CSF (log_2_ transformed). FC: fold change. Data are based on lumbar CSF from 19 subjects and cisternal CSF from 11 subjects.

To identify individual up- or downregulated lipids in lumbar CSF compared to cisternal CSF, the data were subsequently arranged in a volcano plot. Of the 244 detected lipids (Supplementary Table S1, Sheet 1), only eight lipids (PC 32:0, PC(16:0/16:0), PC 31:0, PC 30:0, ethyl oleate, plasmenyl-PE 43:6 and plasmenyl-PE 37:1) were elevated in lumbar CSF compared to cisternal CSF, whereas 190 lipids were significantly elevated in cisternal CSF compared to lumbar CSF (Figure 2, Supplementary Table S1, Sheet 2). The latter belonged to the lipid classes of amides, ceramides, cholesteryl esters, fatty acids, lysophosphatidylcholines, monoacylglycerols, phosphatidic acids, phosphatidylcholines, phosphatidylethanolamines, phosphatidylserines, phosphocholines, plasmenylphosphatidylcholines, plasmenylphosphatidylethanolamines, platelet-activating factors, sphingomyelines, triacylglycerols, and small group collection. Determination of the lipid content within each lipid class in isolation unveiled increased levels of select lipids within four of the main lipid classes in lumbar CSF compared to cisternal CSF (phosphatidylcholines, fatty acids, amides and plasmenylphosphatidylethanolamines) with the elevation of a range of lipids representing most lipid classes in the cisternal CSF compared to lumbar CSF (Figure 3).

## 4. Discussion

Our determination of the lipid composition of CSF across distinct CSF compartments demonstrated differential lipid profiles along the neuroaxis. Lumbar CSF samples are routinely employed in diagnostic workup for individuals presenting with a broad range of neurological diseases offering invaluable insights into CSF composition in common neuropathologies [7,19,22,23,33,34,35,36]. The specific molecular composition of CSF in various pathologies may be employed as a biomarker for a given diagnosis and has, until now, predominantly focused on proteomics, metabolomics, and inflammatory markers, with lipidomics receiving comparatively less attention. Abnormal CSF lipid profiles have been reported in medulloblastoma-related hydrocephalus, in Alzheimer’s disease and in subarachnoid hemorrhage [4,37,38]. In the continued search for biomarkers indicative of various neuropathologies, lipids could thus potentially become useful. However, it is unclear whether lipid biomarkers identified in lumbar CSF reliably signify an intracranial pathology, as the lipid profile along the neuroaxis has remained unresolved. To address this, we employed advanced mass spectroscopy-based lipid quantification of CSF sampled from healthy individuals, either from diagnostic lumbar punctures or from the basal cisterns during the preventive clipping of an unruptured aneurism. We detected the presence of 358 lipids in the CSF distributed across 17 lipid classes, with lipids from most of these lipid classes downregulated in lumbar CSF compared to cisternal CSF. However, six lipids belonging to the classes of fatty acids, amides, phosphatidylcholines, and plasmenylphosphatidylethanolamines were upregulated in lumbar CSF compared to that of the cisterns. Notably, although the lipid concentration was, in general, lower in lumbar CSF, the relative distribution of the lipid classes was distinctly different between the two tested compartments. Such distinct lipid profiles of CSF from the ventricular and lumbar compartments underscore the importance of understanding these differences when utilizing lumbar CSF as a proxy for the CSF bathing the brain tissue and the need for nuanced interpretations when extrapolating biomarker data from lumbar CSF. In comparison, earlier studies have demonstrated similar gradients of various protein biomarkers in CSF along the neuroaxis with some of higher concentration in lumbar CSF and some opposite [25,26,27,39].

The observed difference in lipid composition between CSF in the cisterns and the spinal cord may arise from several factors related to the physiology and dynamics of CSF circulation [40,41,42]. While CSF from the cisterns eventually drains into the spinal cord, the mixing of CSF from various CSF compartments may not be uniform [40]. Additionally, the specific microenvironment within the cisterns, including interactions with neighboring structures and cell types, may influence lipid metabolism and composition [43]. As such, the cisternal CSF, located closer to the brain parenchyma, may have distinct metabolic and biochemical characteristics compared to CSF that has circulated further along the subarachnoid space to the spinal canal [44,45,46,47]. Metabolically, the choroid plexuses in the ventricles could possess their own metabolism and a possible ability to use lipids as a source of energy for their metabolic functions [48]. The lipid metabolism within the central nervous system is highly dynamic and regulated by various enzymatic pathways and transport mechanisms, as the brain itself has a poor capacity to synthetize lipids [49,50,51,52]. These are therefore mainly supplied from peripheral blood circulation [49,50,52,53]. The choroid plexus may selectively regulate the passage of lipids and other molecules into CSF [54]. For example, the ATP-binding cassette transporter, ABCA1, also known as the cholesterol efflux regulatory protein, regulates the release of apolipoproteins by the choroid plexus and hence plays an important role in regulating the transport of cholesterol from the peripheral blood circulation into the CSF [55,56]. Overall, the non-uniform distribution of lipids within the CSF compartments likely reflects the complex interplay of physiological, metabolic, and anatomical factors governing CSF circulation and lipid homeostasis within the central nervous system.

## 5. Limitations

Limitations of the present study include the limited number of patient samples (*n* = 11–19) and uneven distribution in sex (lumbar CSF was sampled from 7F/12M and cisternal CSF from 7F/4M) in addition to the lack of lipid analysis of matched blood samples. Most importantly, the cisternal and lumbar CSF samples were not obtained from the same individuals as dictated by ethical limitations in invasive CSF sampling. Due to statistical requirements of correction for multiple testing necessitated by the extensive quantification of CSF lipids in an untargeted manner, there is a possibility of encountering false negatives. With the untargeted nature of the lipid profiling here employed, we were unable to annotate potential lipid glycosylation and oxidization, perform full analysis of fatty acid components, and detect hydroxy groups, which could all be potential future components to analyze in CSF lipids.

## 6. Conclusions

Our results illustrate that CSF lipids exhibit distinct distribution patterns along the neuroaxis. Consequently, lipid analysis of diagnostically sampled lumbar CSF may not represent the lipid content of intracranial CSF, which could complicate the elucidation of lipid-related etiologies and diagnosis of brain pathologies. Our data provide a first approximation of such a lumbar CSF lipid index. Our findings highlight the need for tailored analytical approaches in CSF biomarker research to refine diagnostic strategies for neurological conditions.

## Data Availability

Data from the study can be provided upon reasonable request. Scripts for the data analysis can be found at https://github.com/Sorennorge/MacAulayLab-Cisternal-Lumbar-Metabolomics.

## Supplementary Materials

The following supporting information can be downloaded at https://www.mdpi.com/article/10.3390/biom14111431/s1, Figure S1: Lipid distribution in cisternal-lumbar, and quality check CSF; Figure S2: Cisternal and lumbar CSF lipid distribution; Table S1: Lumbar and cisternal CSF.
