# Supplementary figures and images for: Differential Lipid Signatures of Lumbar and Cisternal Cerebrospinal Fluid
Source: Biomolecules. 2024 Nov 11;14(11):1431. doi: 10.3390/biom14111431 (PMC11591603; doi:10.3390/biom14111431)

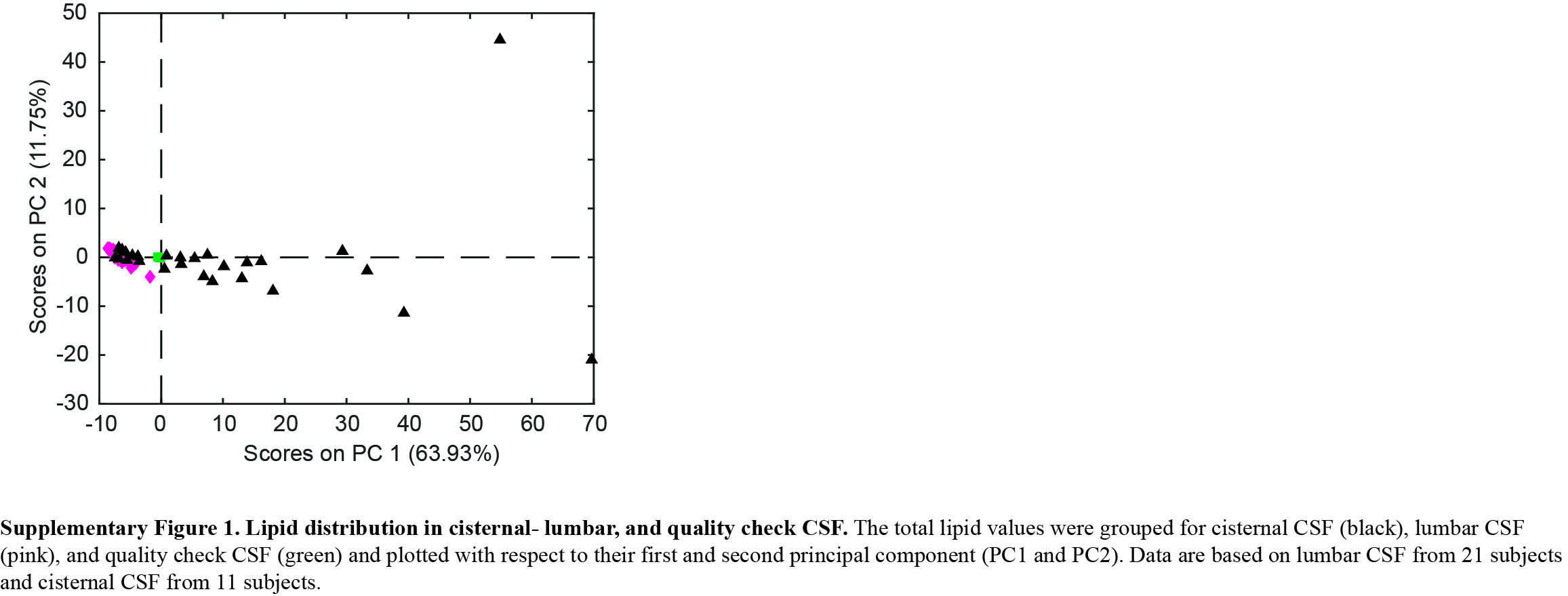

Supplement: Supplementary file 1 [file biomolecules-14-01431-s001.zip › Supplementary Figure S1.jpg]

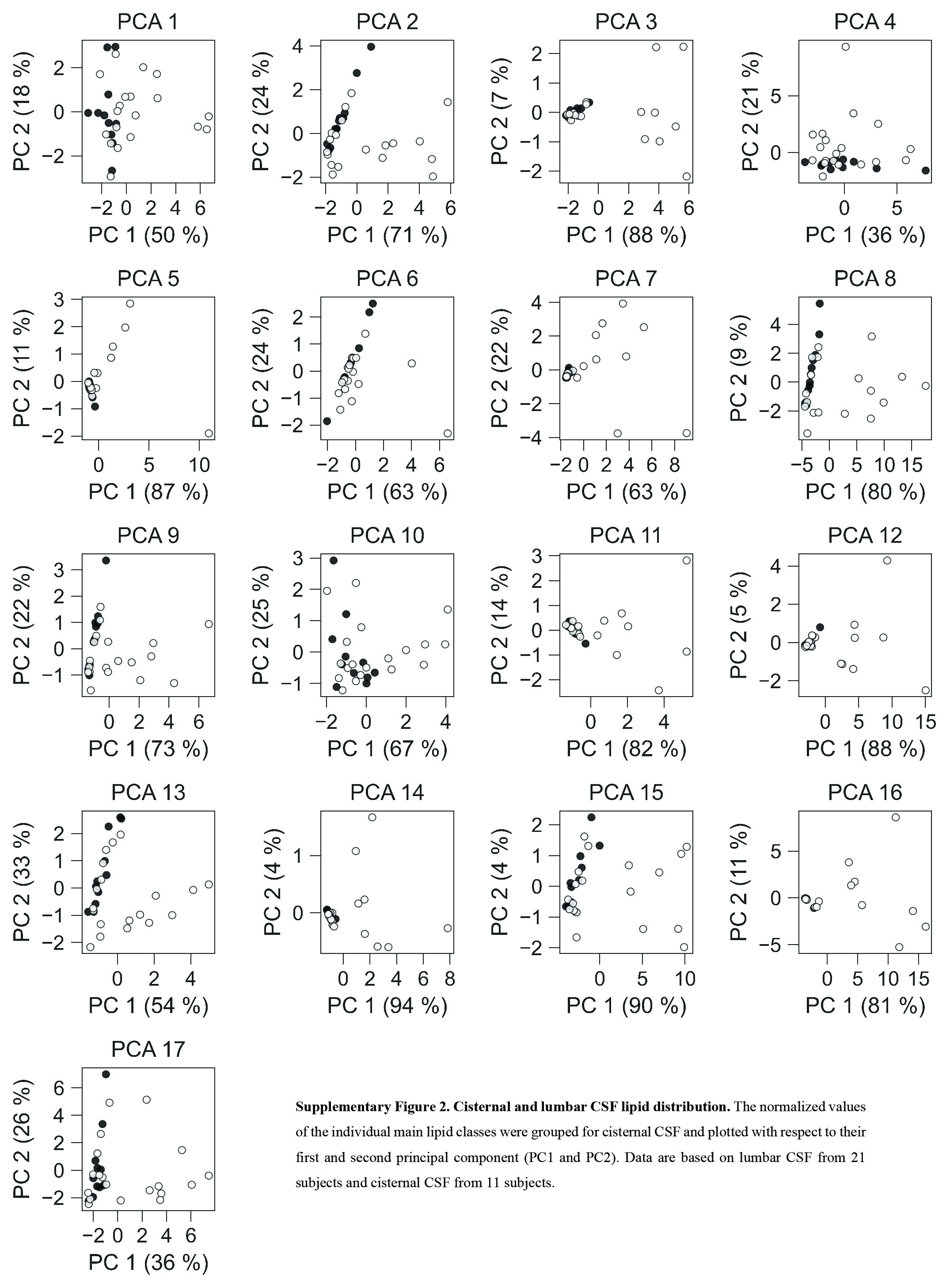

Supplement: Supplementary file 1 [file biomolecules-14-01431-s001.zip › Supplementary Figure S2.jpg]
